# Multiphoton Laser Tomography and Fluorescence Lifetime Imaging of Melanoma: Morphologic Features and Quantitative Data for Sensitive and Specific Non-Invasive Diagnostics

**DOI:** 10.1371/journal.pone.0070682

**Published:** 2013-07-26

**Authors:** Stefania Seidenari, Federica Arginelli, Christopher Dunsby, Paul M. W. French, Karsten König, Cristina Magnoni, Clifford Talbot, Giovanni Ponti

**Affiliations:** 1 Department of Dermatology, University of Modena and Reggio Emilia, Modena, Italy; 2 Department of Physics, Imperial College London, South Kensington Campus, London, United Kingdom; 3 Department of Biophotonics and Lasertechnology, Saarland University, Saarbrücken, Germany; 4 JenLab GmbH, Jena, Germany; 5 Department of Clinical and Diagnostic Medicine and Public Health, University Hospital of Modena and Reggio Emilia, Modena, Italy; The Beatson Institute for Cancer Research, United Kingdom

## Abstract

Multiphoton laser tomography (MPT) combined with fluorescence lifetime imaging (FLIM) is a non-invasive imaging technique, based on the study of fluorescence decay times of naturally occurring fluorescent molecules, enabling a non-invasive investigation of the skin with subcellular resolution. The aim of this retrospective observational *ex vivo* study, was to characterize melanoma both from a morphologic and a quantitative point of view, attaining an improvement in the diagnostic accuracy with respect to dermoscopy. In the training phase, thirty parameters, comprising both cytological descriptors and architectural aspects, were identified. The training set included 6 melanomas with a mean Breslow thickness±S.D. of 0.89±0.48 mm. In the test phase, these parameters were blindly evaluated on a test data set consisting of 25 melanomas, 50 nevi and 50 basal cell carcinomas. Melanomas in the test phase comprised 8 *in situ* lesions and had a mean thickness±S.D. of 0.77±1.2 mm. Moreover, quantitative FLIM data were calculated for special areas of interest. Melanoma was characterized by the presence of atypical short lifetime cells and architectural disorder, in contrast to nevi presenting typical cells and a regular histoarchitecture. Sensitivity and specificity values for melanoma diagnosis were 100% and 98%, respectively, whereas dermoscopy achieved the same sensitivity, but a lower specificity (82%). Mean fluorescence lifetime values of melanocytic cells did not vary between melanomas and nevi, but significantly differed from those referring to basal cell carcinoma enabling a differential diagnosis based on quantitative data. Data from prospective preoperative trials are needed to confirm if MPT/FLIM could increase diagnostic specificity and thus reduce unnecessary surgical excisions.

## Introduction

Melanoma (MM) is a lethal cancer among skin malignancies, with an increasing incidence worldwide [1], [2]. Despite a high cure rate for thin melanomas, advanced melanomas have a poor prognosis. Thus, early identification of melanoma represents a crucial end point for physicians. To optimize early diagnosis, instrumental methods have been introduced to support clinical diagnosis [3]–[5]. However, with established techniques, diagnostic accuracy does not reach 100% [6]. Therefore, further technological efforts are underway with the aim of facilitating the precocious recognition of the black cancer.

**Figure 1:**
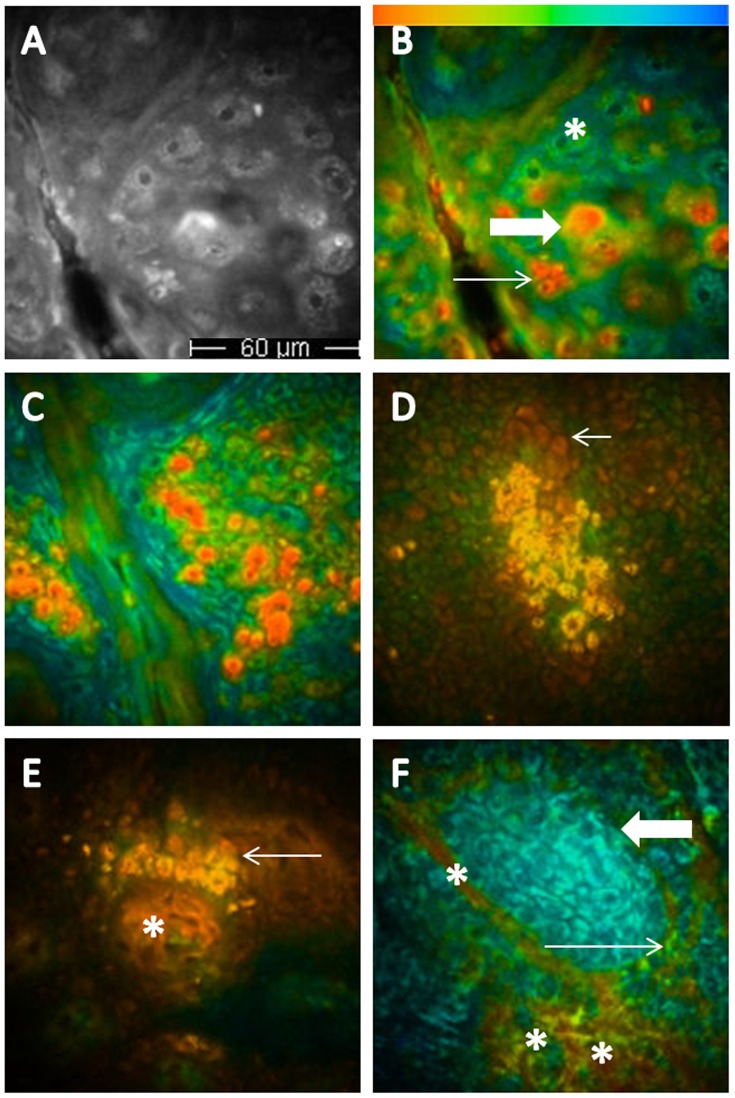
Multiphoton laser tomography and fluorescence lifetime imaging of healthy skin, melanocytic lesions and basal cell carcinoma. A and B. Images referring to the same epidermal area (healthy skin of a phototype 4 subject); in A, the multiphoton image, in B, the corresponding color coded image, enabling the recognition of green large keratinocytes (keratinocytes without pigment, asterisk), large orange melanin-containing keratinocytes (thick arrow) and small red melanocytes (thin arrow). C, D and E. Melanocytic nevus; C, red-orange cells corresponding to melanocytes, regular in size and shape in the upper epidermal layers; D, a melanocytic nest, consisting of aggregated orange melanin containing cells at the dermal-epidermal junction (the arrows indicate the papillae); E, a small melanocytic nest close to the tip of the papilla (arrow), where red collagen fibers are visible (asterisk). F, basal cell carcinoma; a nest of blue basaloid cells is clearly visible (thick arrow), infiltrating green normal keratinocytes (thin arrow); asterisks indicate blood vessels.

Multiphoton excited fluorescence imaging (multiphoton tomography, MPT) with near-infrared femtosecond-pulsed lasers enables ex vivo and in vivo non-invasive investigation of the skin with subcellular resolution (0.5 µm in lateral direction and 1–2 µm in the axial direction) [7]–[11]. Due to naturally occurring endogenous fluorescent bio-molecules such as flavins, NAD(P)H coenzymes, metal-free porphyrins, components of lipofuscin, melanin, elastin, collagen and keratin, fluorescence imaging can be performed without additional contrast agents. Fluorescence light emitted by organic molecules is not only characterized by its emission intensity, but also by the shape of the fluorescence emission spectra [12] and by specific fluorescence decay times [13], [14], which can also be influenced by the metabolic state of the cells [15], [16]. Fluorescence lifetime imaging (FLIM) can be performed using fast detection electronics [13], and the resulting data is usually presented using a false-color scale image where color encodes fluorescence lifetime. Since melanin exhibits a short fluorescence decay component of around 200 ps [17], FLIM can be used to distinguish cells containing melanin from keratinocytes that do not contain melanin and exhibit predominantly NAD(P)H fluorescence with a longer mean fluorescence lifetime (∼0.4–2 ns depending on protein binding state) [18], [19] (Fig. 1). Thus, FLIM is particularly suited for the study of melanocytic lesions [18], [20]. With MPT-FLIM, bidimensional images are acquired and correspond to optical sectioning parallel to the tissue surface. Pictures obtained at various depths are generated by sequentially modifying the depth of the focus plane in the tissue reaching levels of 200 µm. Thus, only the superficial part of skin tumors can be studied.

The aim of our study was to identify morphologic descriptors for melanoma, referring both to cellular features and to architectural aspects, employing MPT fluorescence intensity and FLIM (MPT/FLIM descriptors), and to establish a specific terminology for their assessment. Subsequently, we evaluated the frequency of these descriptors in 3 different populations comprising melanomas, melanocytic nevi and basal cell carcinomas (BCCs), to establish sensitivity and specificity of MPT/FLIM diagnosis. Finally, the diagnostic accuracy for MM achieved by MPT/FLIM was compared to that of dermoscopy.

## Materials and Methods

### Study Design

The study was divided into two parts, a training phase and a test phase. In the former, FLIM images referring to 6 MMs were simultaneously evaluated by 3 observers (SS, CD, PF), for the identification of morphologic descriptors characteristic of MM. Proposed descriptors were discussed and 30 parameters were selected after unanimous approval.

In the test phase, the presence/absence of the specified criteria were blindly evaluated on a test data set, by 3 independent observers (SS, FA, CM), who also classified the images into those pertaining to melanoma or "other".

Moreover, the mean fluorescence lifetime inside a region of interest corresponding to one representative melanin containing cell of each sample, was calculated on images acquired at an excitation wavelength of 760 nm on 25 MMs, 50 nevi and 50 BCCs samples.

The local ethics committee (University of Modena and Reggio Emilia, Modena University Hospital Ethics Committee) granted approval of the study protocol before the start of the study. The patients provided their written informed consent.

### Patients and Lesions

The training set included 6 melanomas with a mean Breslow thickness±S.D. of 0.89±0.48 mm. In the test phase *ex vivo* samples of 25 MMs, 50 nevi and 50 BCCs were considered. MMs, of the superficial spreading type, included 8 in situ lesions and had a mean Breslow thickness (± S.D.) of 0.77 (±1.2) mm (thickness range 0–4.7 mm). No amelanotic and nodular melanomas or lesions showing histological regression were included, but 3 MMs showed a nodular component. Nevi were removed surgically because of equivocal dermoscopic features. BCCs comprised 31 nodular BCCs and 19 superficial BCCs, and one third were pigmented. In all cases, the clinical diagnosis was confirmed by conventional histopathological examination.

### Instrumentation and Elaboration of the Data

#### Multiphoton tomography

In this study we used the MPT DermaInspect® imaging system (Jenlab GmbH, Jena, Germany), which provides intra tissue scanning with subcellular spatial resolution (0.5 µm in lateral direction and 1–2 µm in the axial direction) [8], [9], [18]. The instrument has been described in detail elsewhere [19], [20].

#### Fluorescence lifetime imaging

FLIM was implemented in the DermaInspect® system using a TCSPC module with a temporal instrument response function of approximately 250 ps (full-width at half-maximum). Data was collected with time bins separated by 50 ps. A short pass optical filter (Schott BG39) was used to protect the detector (PMH 100-1, Becker & Hickl GmbH, Berlin, Germany) from scattered excitation light. The resulting spectral detection window was ∼ 340 nm –610 nm.

**Figure 2:**
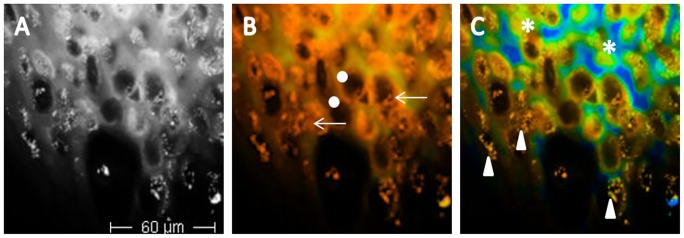
Multiphoton laser tomography and fluorescence lifetime imaging of melanoma. A, MPT image of upper melanoma layers; B, FLIM image of the same spot displayed by a 0–2000 *ps* fluorescence lifetime range showing that all cells are melanocytic; C, 0–400 scale, employed to increase contrast for the distinction of cellular details, such as the cytoplasmic halo and the speckling. Dots indicate nuclei with undefined contours; arrows indicate polinucleated cells; asterisks indicate cytoplasmic halo, and triangles speckled melanin. FLIM values do not change, they are only displayed in a different way.

The FLIM images were calculated using the software SPCImage (Becker & Hickl GmbH). The raw data was smoothed using a 5 x 5 kernel (corresponding to binning setting 2 in SPCImage) and a single exponential decay was fitted at each pixel. The final image output of the FLIM analysis is a pseudocolor image where the color scale encodes the fluorescence lifetime and image brightness encodes the fluorescence intensity. Images were displayed with a red to blue color scale corresponding to 2 fluorescence lifetime ranges: 0–2000 ps (all figures except Fig. 2c) and 0–400 ps (Fig. 2c). We note that the fluorescence decay recorded in a given single pixel can originate from multiple autofluorescent species that can each have complex multi-exponential decay profiles. Given the relatively small number of photons detected in a single pixel, we chose to fit a single exponential decay model to the data in order to generate an estimate of the mean fluorescence lifetime in that pixel. While more complex multi-exponential decay models can provide additional information, e.g. on relative contributions from different fluorescent species, the use of a single exponential decay model is a straightforward approach enabling fluorescence lifetime contrast to be extracted from the fluorescence decay data.

### Image Acquisition and Analysis

Ex vivo samples were placed on a cork disc covered by a transparent film and a gauze. For the examination of the samples a drop of water was placed between the skin and the cover glass, which was then attached to a metal coupling ring by circular adhesive tape. The metal coupler was magnetically connected to the MPT system after applying a drop of oil (Immersol™ 518F, Carl Zeiss Ltd, Germany). After finding the correct level, the focus on a specific layer was found by means of the software JLScan_1100. The imaging depth was first pinpointed to the upper layer (stratum corneum). Subsequently, two-dimensional, horizontal images were acquired through optical sectioning parallel to the tissue surface (i.e., in the x–y plane) with an interval of 15 µm. Three-dimensional data sets, called z-stacks, were obtained by sequentially changing the plane of focus (z-level), thus scanning at different tissue depths to a depth of approximately 200 µm.

One optical section, consisting of 256×256 pixels, was taken with a scanning time of 25.5 s/frame and an excitation power between 20 and 45 mW. Excitation wavelengths of 760 nm and 820 nm were applied for the assessment of the lesions.

When imaging in the dermis, autofluorescence signals from both collagen fibers (SHG signal) and elastin fibers (autofluorescence) can be generated. Separation of these signals was accomplished by an optical filter (Semrock, BLP01-405R-25, ∼0.04% transmission at 415 nm) that can be inserted into the beam path and which excludes the SHG component of the signal when exciting at up to 820 nm. Imaging was accomplished within 30 minutes from biopsy.

FLIM images produced by SPCImage were analysed using a custom-written software package to calculate the mean fluorescence lifetime values for selected areas of interest such as single cells on 25 MMs, 50 nevi and 50 BCCs.

### Dermoscopy

Dermoscopic diagnosis was performed on 20-fold and 50-fold magnified images acquired by means of a digital epiluminescence microscope (FotoFinder, TeachScreen software GmbH, Bad Birnbach, Germany) by 3 observers experienced in dermoscopy according to established dermoscopic criteria for MM [22]–[27].

The test data set for dermoscopy comprised the same lesions studied by MPT/FLIM (25 MMs, 50 nevi and 50 BCCs). Images were presented in a mixed order to the observers, who evaluated them blinded to the histopathologic diagnosis and without knowledge of any clinical data of patients and lesions. For each lesion, observers were asked to make the diagnosis of MM vs non MM. In the absence of unanimity, the diagnosis was established when 2 out of the 3 observers agreed.

### Statistics

The frequency of each descriptor in MM, nevi and BCC samples was computed. Sensitivity and specificity values of single descriptors and of the MPT/FLIM diagnosis of MM were evaluated as compared with histopathologic diagnosis. Moreover, odds ratios (OD) were calculated for each descriptor. The agreement between ratings made by 3 observers on the diagnosis and specific patterns of MM (inter-rater reliability) was estimated using Cohen kappa statistics with 95% confidence intervals. Finally, mean values and standard deviations of the fluorescence decay in MM, nevus and BCC cells were calculated and differences were assessed by means of the *t*-test. A *p*-value <0.05 was considered statistically significant. Statistical examinations were performed with SPSS 19.0 for Windows (SPSS Inc., Chicago, IL, U.S.A.).

## Results

All image analysis was performed using pseudocolor FLIM images produced by the SPCImage software. For each sample, 15 FLIM images were examined. In total, 375 images of MM, 750 images of nevi and 750 images of BCC were considered.

### Training Phase

#### Identification of MPT/FLIM descriptors for melanoma and choice of the appropriate terminology (Table 1)

Considering 90 images corresponding to 6 MM stacks, 30 descriptors were identified. Keratinocytes, which are not always recognizable at the surface, are infiltrated by atypical melanocytes. When displaying images using a 0–2000 ps fluorescence lifetime range, MM cells exhibit a short fluorescence decay time (red on false-colour scale); thus, a carpet of red cells appears at the lesion surface. Based on previous published work [18], we attribute this short fluorescence lifetime to a high concentration of melanin (with a dominant short 200 ps decay component) relative to NAD(P)H (mean fluorescence lifetime ∼0.4–2 ns depending on protein binding state), and this immediately enables the determination of the melanocytic nature of the lesion (Fig. 2b).

**Figure 3:**
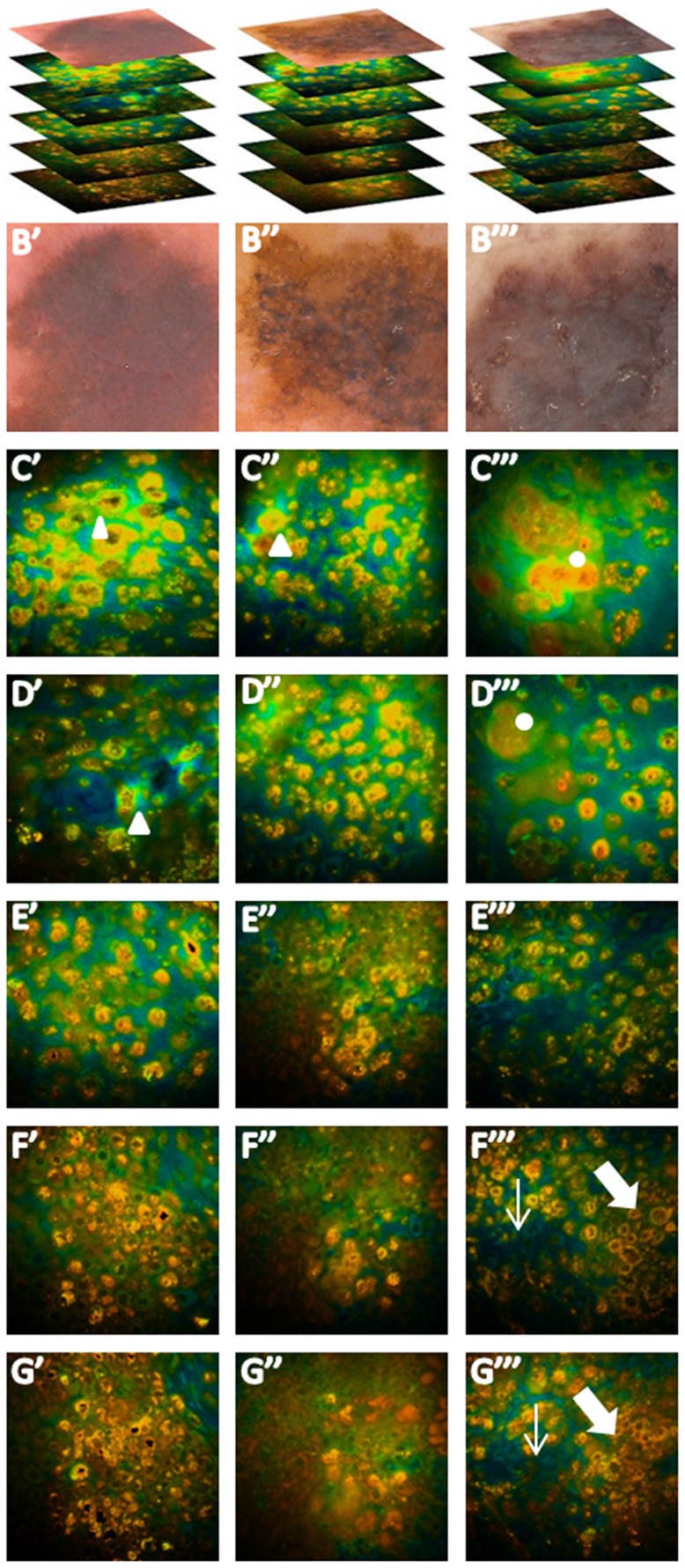
Fluorescence lifetime imaging of melanoma at different depth. A, dermoscopic images and stacks of 3 melanomas; B, typical dermoscopic features; C, and D, upper melanoma layers, characterized by orange atypical large short-lifetime cells (ASLCs) irregularly distributed or forming aggregates. Cells are pleomorphic and show a nucleus with undefined contours and a non-homogeneous cytoplasm with speckled melanin. A peripheral cytoplasmic halo is observable in some cells (triangles), some of which are polinucleated (dots). E, F and G, deeper MM layers with ASLCs variable in size, shape and distribution forming aggregates and infiltrating papillae (thick arrows) and hair follicles (thin arrows).

**Figure 4:**
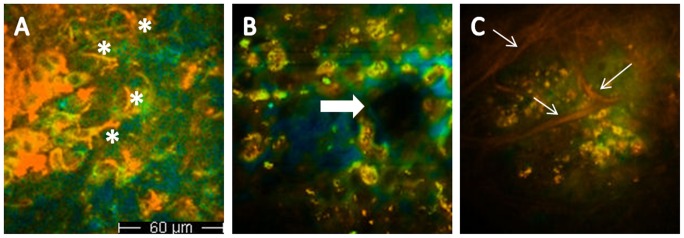
Fluorescence lifetime imaging: different features of melanoma. A, dendritic cells in upper melanoma layers (asterisks); B, melanoma cells infiltrating a non edged papilla (arrows); C, red collagen fibers surrounding ASLC nests (arrows).

To increase contrast for the distinction of image details, a 0–400 ps scale can be applied (Fig. 2c). Since in MM the epidermal architecture is completely disrupted, it is not possible to refer to epidermal layers for the description of the morphology of MM. Thus, a description of MPT/FLIM aspects of MM was performed considering 2 different levels: a superficial level consisting of the upper 30 µm and an inferior one comprising all the lower layers (Fig. 3). At the surface, atypical cells appear as large short-lifetime cells (ASLCs) of different size, with a nucleus with undefined contours and a non-homogeneous cytoplasm with speckled melanin (Fig. 3c,d,e). A peripheral cytoplasmic halo is often observable due to variable lifetime values of different cell areas. MM cells may show more than one nucleus. Besides large cells, smaller cells of different size can be seen. They may be irregularly distributed or may form aggregates. When the epidermal structure is still partially observable, ASLCs infiltrate the keratinocytes. When present, dendritic cells are easily recognized by their ramifications (Fig. 4a). In some cases, long lifetime cell nests appearing as aggregates of blue-green spindle shaped cells (probably corresponding to melanocytic cells without melanin) are observable.

Deeper MM layers show ASLCs smaller in size with respect to those observable at the surface (Fig. 3f,g). They are pleomorphic, with variable size and irregular shape and show an irregular distribution. At this level ASLCs may form aggregates appearing as sheets or nests and may infiltrate dermal papillae and hair follicles (Fig. 4b). When shifting the excitation wavelength to 800–820 nm, to explore the extracellular matrix, collagen fibers are seldom visible; in the example shown in Fig. 4c, they surround ASLC nests. When dermal papillae are recognizable, they are mostly not surrounded by a rim of regularly arranged melanocytes as in melanocytic nevi [20], show irregular size and shape and are separated by interpapillary spaces of different thickness (non-edged papillae). An architectural disorder is generally observable.

### Testing Phase

#### Frequency of MM descriptors (Table 1)

The frequency of MM descriptors in MMs, nevi and BCCs is illustrated in Table 1. The frequency of MM descriptors for MM ranged between 96% and 8%. ASLCs in the upper layers were present in 24 out of 25 MMs, and were characterized in most cases by large cells (88%), different in size (88%) with a nucleus with undefined contours (92%) and speckled melanin (88%). One third of these cells showed a cytoplasm with a halo; 76% were polinucleated, and 92% were irregularly distributed. Aggregates of these atypical cells were found in 19 MMs, whereas in 15 MMs ASLCs infiltrating epidermal cells were recognizable at the surface. In nevi, keratinocytes were always present at the surface, and in 18% of the cases only, some atypical cells were visible. However, these never showed more than one nucleus and a cytoplasmic halo. Sporadic short lifetime cells, large and different in size, were also visible in 3 pigmented BCCs, but they never showed other features characterizing ASLCs in melanocytic lesions. We were able to detect dendritic cells in 5 MMs, 2 nevi and one BCC. The characteristics of these cells did not differ between the 3 populations.

**Table 1:** Frequency of MPM/FLIM descriptors in 25 MMs, 50 melanocytic nevi and 50 BCCs.

MPT/FLIM descriptors	MMs N°	MMs % (sens.)	Nevi N°	Nevi %	BCCs N°	BCCs %	Specificity	Cohen's k coefficient range	X^ 2^	Oddsratios	Upper confidenceinterval	Lower Confidenceinterval	
Upper layers													
Keratinocytes (KCs) at the surface	15	60	50	100	15	30	0	0.884–1.000	23.077*	NA	NA	NA	
Atypical short lifetime cells (ASLC) present	24	96	9	18	3 **	6	82	NA	45.221*	NA	NA	NA	
ASLC large	22	88	9	18	0	0	82	0.468–1.000	41.153*	109.333	13.038	916.820	
ASLC different size	22	88	9	18	3	6	82	0.468–1.000	41,153*	109.333	13.038	916.820	
ASLC nucleus with undefined contours	23	92	9	18	0	0	82	0.648–1.000	41.153*	109.333	13.038	916.820	
ASLC speckled melanin	22	88	4	8	0	0	92	0.884–1.000	47.096*	84.333	17.356	409.768	
ASLC non homogeneous cytoplasm	19	76	2	4	0	0	96	0.884–1.000	42.857*	76.000	14.077	410.308	
ASLC halo cytoplasm	8	32	0	0	0	0	100	0.884–1.000	17.910*	NA	NA	NA	
ASLC polinucleated	19	76	0	0	0	0	100	0.884–1.000	50.893*	NA	NA	NA	
ASLC irregular distribution	23	92	1	2	0	0	98	0.648–1.000	66.270*	1176.000	70.484	19621.241	
ASLC aggregates	19	76	0	0	0	0	100	0.884–1.000	50.893*	NA	NA	NA	
ASLC infiltrating KCs	15	60	0	0	0	0	100	0.884–1.000	50.893*	NA	NA	NA	
Dendritic cells	5	20	2	4	1	2	96	0.884–1.000	6.996*	7.579	1.404	40.917	
Long lifetime cell nests	3	12	0	0	0	0	100	0.503–1.000	8.451*	NA	NA	NA	
Lower layers													
ASLC	22	88	2	4	0	0	96	0.468–1.000	62.284*	576.000	49.707	6674.587	
ASLC different size	22	88	1	2	0	0	98	0.468–1.000	66.270*	1176.000	70.484	19621.241	
ASLC nucleus with different size	19	76	0	0	0	0	100	0.884–1.000	50.893*	NA	NA	NA	
ASLC irregular shape	21	84	0	0	0	0	100	0.884–1.000	58.333*	NA	NA	NA	
ASLC irregular distribution	21	84	1	2	0	0	98	0.843–1.000	57.974*	359.333	35.370	3650.594	
ASLC aggregates	18	72	0	0	0	0	100	0.884–1.000	47.368*	NA	NA	NA	
ASLC sheets	21	84	0	0	0	0	100	0.884–1.000	58.333*	NA	NA	NA	
ASLC nests	8	32	0	0	0	0	100	0.905–1.000	15.441*	NA	NA	NA	
ASLC invading the papillae	13	52	0	0	0	0	100	0.920–1.000	28.571*	NA	NA	NA	
Nests and fibers	3	12	0	0	30	60	100	0.884–1.000	6.250*	NA	NA	NA	
Cells and fibers	2	8	0	0	0	0	100	0.884–1.000	4.110*	NA	NA	NA	
Architecture													
Epidermal structures recognizable	11	44	50	100	15	30	0	0.759–1.000	27.444*	0.019	0.002	0.158	
Architectural disorder	24	96	2	4	50	100	96	0.884–1.000	62.284*	576.000	49.707	6674.587	
Non edged papillae	10	40	5	10	0	0	90	0.746–1.000	7.421*	5.063	1.475	17.374	
Edged papillae	3	12	50	100	0	0	0	0.519–1.000	43.174*	0.008	0.001	0.069	
No recognizable papillae	15	60	0	0	50	100	100	0.690–1.000	34.426*	NA	NA	NA	
Diagnosis of melanoma by MPT/FLIM	25	100	1	2	0	0	98	–	–	–	–	–	

Cohen's k coefficients of single descriptors, X^2^ values and Odd's ratios in 25 MMs and 50 nevi.

• = X^ 2^ significancy (*p*≤0.005). The value is NA (not applicable) when it was not possible to find: nevi with a positive parameter, nevi with a negative parameter, melanomas with a positive parameter, melanomas with a negative parameter.

** Melanin containing cells in 3 BCCs were large and with different size, but did not show all the atypical aspects of melanoma cells.

Lower MM layers were characterized by ASLCs, different in size, in 88% of the cases. These frequently showed nuclei of different size, were irregular in shape and irregularly distributed, forming aggregates (72%), sheets (84%) or nests (32%). Invasion of the papillae was visible in half of the cases, whereas nests surrounded by fibers or single ASLCs intermingled with fibers were observable in approximately 10% of the cases. Short lifetime cells, different in size or with an irregular distribution were found in 2 nevi.

With regard to structural aspects, an architectural disorder was observed in 24 MMs and in 2 nevi. Non edged papillae were observed in 40% of MMs and in 10% of nevi. These were always associated to edged papillae in nevi, whereas in MMs, edged papillae were recognizable only in 3 cases. The χ square test, calculated on the populations of MMs vs nevi, was significant for all descriptors.

#### Sensitivity and specificity for MM diagnosis and for single MM descriptors

The classification of a lesion as MM was made based on the presence of ASLCs in the upper layers and architectural disorder. This approach correctly identified 100% of the cases of MM and produced one false positive in nevi (Table 1). Sensitivity and specificity for single MM descriptors were high for most descriptors.

#### Interobserver agreement (Table 1)

The results of the kappa statistics analysis showed a good reliability of all criteria with kappa values ranging from 0.468 to 1.000.

#### Odds ratios (Table 1)

Among the descriptors, the ones with the highest probability to identify a MM (OR  = 1176) were "ASLCs irregular distribution upper layers" and "ASLCs different size lower layers", followed by "ASLCs lower layers" and "architectural disorder" (OR  = 576).

#### Mean fluorescence lifetime values (Table 2)

Fluorescence lifetime values obtained from a single exponential decay fit to the data (+ s.d.), calculated on 3 representative cells on both upper and lower layers of 25 MMs were 347+159 ps and 338+170 ps, respectively. No difference was observed between these values and those referring to nevi (362+121 ps), whereas for BCC the fluorescence lifetime value was significantly higher (1395+130 ps).”

**Table 2:** Fluorescence decay value of melanomas, melanocytic nevi and basal cell carcinomas.

Fluorescence decay value	MMs mean and s.d.	Nevi mean and s.d.	BCCs mean and s.d.
Upper layers	347.23±158.82	362.06±120.71	1395.25±129.82
Lower layers	338.37±169.75	–	–

#### Dermoscopy

All MMs were correctly diagnosed by dermoscopy (sensitivity = 100%), whereas 9 nevi were diagnosed as MM (specificity = 82%).

## Discussion

Instrument-based methods have been introduced as a support to clinical diagnosis for early detection of MM and improvement of its prognosis. Dermoscopy is nowadays widely employed in clinical practice [28]. Recently, reflectance confocal microscopy (RCM) has been introduced in selected settings as an imaging method that allows real-time skin examination at a subcellular resolution, further improving melanoma diagnosis [29], [30]. As a technique with a resolution superior to that of RCM, MPT constitutes one of the most promising and fastest developing technologies in the field of optically sectioned clinical imaging. It is based on the emission of naturally occurring endogenous fluorescent bio-molecules. These, however, often present overlapping fluorescence spectra, thus hampering the distinction between different fluorophores or metabolic states.

Melanins are the main pigments of both constitutive and light-induced human skin color. They are synthesized in specialized organelles and then transferred to surrounding keratinocytes. Melanin content is generally increased in benign and malignant melanocytic lesions and may represent a clue to their recognition. Melanins differ from other skin fluorophores owing to their short fluorescence decay component. Dimitrow et al. investigated procedures of spectral FLIM on 46 melanocytic lesions, and observed remarkable differences in lifetime behaviour between keratinocytes and melanocytes [18]. Fluorescence lifetime distribution varied according to the intracellular amount of melanin. However, FLIM on single data points seemed to be unsuitable for a classification of benign versus malign melanocytic skin lesions. Thus, only the combination of morphologic features with lifetime behavior studies employing MPT associated to FLIM provides clues to cell differentiation.

Distinct morphological differences between MM and melanocytic nevi were detected in a study employing only MPT fluorescence intensity imaging by the same group considering 83 melanocytic skin lesions including 26 MMs [21]. Six characteristic features of MM were described, and, among these, architectural disarray of the epidermis, poorly defined keratinocyte cell borders and the presence of pleomorphic or dendritic cells were of prime importance for the diagnosis.

In order to assess the potential use of the combination of MPT with an improved FLIM technique in MM diagnosis, we performed an observer-blinded study on melanocytic skin lesions to identify robust MPT/FLIM descriptors enabling diagnostic sensitivity, specificity, and reliability values, high enough to guarantee clinical applicability and provide results better than those obtained by a conventional imaging technique, i.e. dermoscopy.

MM cells on the surface of the lesion are recognizable at the first glance: starting the scanning of a malignant melanocytic lesion, these pleomorphic atypical cells entirely occupy the upper sheets and are red colored (short lifetime) when employing a 0–2000 ps color scale. ASLCs appear as single cells, irregularly distributed or aggregated, with a distribution similar to that of ascending melanocytes in histopathological sections.

Contrary to what was observed by Dimitrow et al. [21], a residual epidermal structure could be recognized in a minority of MM cases, in contrast to nevi where epidermal structures were always distinguishable [21]. These observations reflect the classic cytological changes detected in histopathologic sections of melanoma, including cellular atypia and pleomorphism, asymmetry of cytological details, and pagetoid spread, i.e. melanocytes scattered in the epidermis and in the follicular epithelium [31]. Our morphological data are also in line with those obtained by RCM, where highly diagnostic features associated with malignancy are the partial or total loss of the honeycomb epidermal pattern and the pagetoid spread of roundish or dendritic cells [29], [30]. By MPT/FLIM, in lower MM layers, atypical cells are smaller and may form aggregates, sheets or nests. At this level the architecture is completely disarranged and papillae lacking a border of well-arranged cells are invaded by atypical melanocytes. These aspects correspond to the presence of atypical cells in the basal layer and irregular or non-edged papillae by confocal [29], [30]. Conversely, a monomorphic and regular histoarchitecture was observed in nevi.

In benign lesions, melanocytes were typically visualized as small cells, and nevus cell nests were found at the dermoepidermal junction. Dendritic cells, representing a major diagnostic criterion for MM diagnosis according to Dimitrow et al., were observable only in one fifth of MM cases and showed the same morphology of those observed in nevi and BCCs. Short lifetime cells, variable in size, were also found in 6% of BCC cases. However, these cells never showed more than one nucleus, a nucleus with undefined contours or a non-homogeneous speckled cytoplasm, like in MMs; they also did not form aggregates and were regularly distributed.

BCCs showed the typical structure characterized by elongated cells with double alignment, nests surrounded by fibers and phantom islands [32]–[34]. Moreover, the corresponding pseudocolor fluorescence lifetime images displayed typical blue colored (longer lifetime) cells with a mean lifetime of 1400 ps that were readily distinguishable from red-coloured (shorter lifetime) cells containing melanin. In previous studies fluorescence lifetimes of healthy epidermal cells in young subjects calculated by our group were around 1100 ps for upper epidermal layers and 900 ps for lower ones, corresponding to the green color [19]. In the epidermis of elderly subjects, keratinocytes show a shift in fluorescence decay times towards higher values with respect to young epidermal cells (blue-green) [19].

Fluorescence lifetime depends not only on the fluorophore itself, but also on the interaction with the local environment [35]. Thus, changes in fluorescence lifetime with cancerization can be due both to alterations of cell proliferation and metabolic pathways and to tissue vascularisation and oxygen supply [36], [37].

Similarly to previous published studies [18], our data did not reveal any difference between mean fluorescence lifetime values of melanocytic cells in nevi and those in MMs. It has been shown that, whereas keratinocyte lifetime values correspond to NAD(P)H [12], [38], [39], melanocyte ones match with the endogenous fluorophore melanin [17], [18]. In the process of malignant transformation of melanocytic lesions, an increase of the ratio of pheomelanin to eumelanin has been reported [40], which has been linked to an increase in fluorescence emission in MM in the red region of the fluorescence emission spectrum [41]–[42]. However, this shift in emission spectrum apparently does not influence the fluorescence decay time, which our results show are similar in malignant and benign melanocytic cells. Thus, when employing our MPT/FLIM instrument, the differentiation between keratinocytic and melanocytic skin tumors can be achieved immediately on the basis of fluorescence decay data, whereas a diagnosis of malignant melanocytic lesion has to be based also on morphologic observations.

Most MPT/FLIM descriptors identified in this study showed high sensitivity and specificity values. Moreover, a sensitivity of 100% and a specificity of 98% were reached for the diagnosis of MM. These values were higher than those reported by Dimitrow et al., which were based on a reduced number of parameters, attaining an overall sensitivity of 84% and an overall specificity of 76%. Moreover, a comparison between the diagnostic capability of MPT/FLIM and that of dermoscopy, which at present represents the established diagnostic technique for MM, was performed for the first time in this study.

Although the penetration depth of MPT is at present limited to 200 µm, making the evaluation of MM thickness impossible, the MPT/FLIM technique holds great promise for in vivo diagnosis of melanoma, enabling a diagnostic accuracy higher than that achieved by dermoscopy. The nevi imaged in this study had been removed surgically because of equivocal dermoscopic aspects and were difficult to diagnose even by an experienced dermoscopist. Thus, MPT/FLIM used in a preoperative phase would have increased diagnostic specificity and reduced unnecessary surgical excisions.
